# Identification of Immune-Related Risk Genes in Osteoarthritis Based on Bioinformatics Analysis and Machine Learning

**DOI:** 10.3390/jpm13020367

**Published:** 2023-02-19

**Authors:** Jintao Xu, Kai Chen, Yaohui Yu, Yishu Wang, Yi Zhu, Xiangjie Zou, Yiqiu Jiang

**Affiliations:** 1 Department of Sports Medicine and Joint Surgery, Nanjing First Hospital, Nanjing Medical University, Nanjing 210000, China; 2 Jiangsu Province Hospital, The First Affiliated Hospital With Nanjing Medical University, Nanjing 210000, China

**Keywords:** WGCNA, osteoarthritis, RNA-seq, disease markers, immune

## Abstract

In this research, we aimed to perform a comprehensive bioinformatic analysis of immune cell infiltration in osteoarthritic cartilage and synovium and identify potential risk genes. Datasets were downloaded from the Gene Expression Omnibus database. We integrated the datasets, removed the batch effects and analyzed immune cell infiltration along with differentially expressed genes (DEGs). Weighted gene co-expression network analysis (WGCNA) was used to identify the positively correlated gene modules. LASSO (least absolute shrinkage and selection operator)-cox regression analysis was performed to screen the characteristic genes. The intersection of the DEGs, characteristic genes and module genes was identified as the risk genes. The WGCNA analysis demonstrates that the blue module was highly correlated and statistically significant as well as enriched in immune-related signaling pathways and biological functions in the KEGG and GO enrichment. LASSO-cox regression analysis screened 11 characteristic genes from the hub genes of the blue module. After the DEG, characteristic gene and immune-related gene datasets were intersected, three genes, PTGS1, HLA-DMB and GPR137B, were identified as the risk genes in this research. In this research, we identified three risk genes related to the immune system in osteoarthritis and provide a feasible approach to drug development in the future.

## 1. Introduction

Osteoarthritis (OA) is a multifactorial disease affecting millions of people worldwide [1]. The gold standard in the clinical management of OA patients is joint replacement surgery in the terminal stage of the disease [2]. Timely intervention is requisite in the early stage of the disease. However, due to the complicated pathogenesis of osteoarthritis [3], there currently is no effective medical treatment except for pain management. Thus, the mechanism of osteoarthritis urgently needs to be clarified.

In recent years, there has been quite some effort in identifying disease markers and predicting the clinical prognosis of osteoarthritis using computational tools. Han et al. first combined WGCNA and immune infiltration in analyzing osteoarthritis-related datasets [4]. Meng et al. made comprehensive use of LASSO regression and SVM-RFE (support vector machine recursive feature elimination) followed by experimental methods and demonstrated the crucial function of PDK1 in regulating the progression of osteoarthritis [5]. Moreover, computational tools also imply a powerful future in terms of predicting disease progression. Janvier etc. reported a new strategy where the progression of knee osteoarthritis could be predicted through assessing the trabecular bone texture in different locations of the knee [6]. Khaled applied use of the Logitboost model in analyzing gray level co-occurrence and local binary patterns to accurately forecast pathological progress [7]. Therefore, computational tools are of great potential in deciphering the secret of osteoarthritis.

The innate immune system has been reported to participate in OA progression [8]. For instance, the macrophages M1 and M2 play different roles in cartilage degradation and regeneration [9]. The T cells secret cytokines and growth factors to impact the extracellular matrix (ECM) [10]. Natural killer (NK) cells exert immunoregulatory effects on the immune response by promoting inflammation [11]. Therefore, it is necessary to determine the expression and distribution of immune cells in osteoarthritis joints.

Synovial lesions contribute to cartilage erosion to a great extent [12]. Immune cells are also significant in synovial inflammation and impact the cartilage through cross-talk [13]. Pro-inflammatory T cells contribute to cartilage matrix degradation from the beginning of the disease [14]. Mast cells can promote inflammation or induce chondrocyte apoptosis in different phases [15]. However, immune cell infiltration differences between the synovium and cartilage have not been studied yet.

In this research, we first combined advanced bioinformatics methods to comprehensively analyze the gene expression and immune cell infiltration in the synovium and cartilage of healthy people and OA patients. Our results for the first time combine datasets from different sources and demonstrate that immune cell infiltration differs significantly in the synovium compared to the cartilage. Collectively, we offer a new strategy in identifying disease markers of osteoarthritis based on focusing prominently on immune-related genes and provide three characteristic genes for future drug development and susceptible population screening.

## 2. Materials and methods

### 2.1. Collection of Datasets

We used the keywords “synovium”, “osteoarthritis” and “cartilage” to screen the appropriate datasets in the Gene Expression Omnibus database (GEO). Finally, we selected GSE55235, GSE55457 and GSE82107 as the datasets for synovium-related analysis and GSE57128, GSE117999 and GSE169077 for cartilage-related analysis. It should be noted that datasets only involving the groups “healthy” and “osteoarthritis” are rare. Thus, we excluded rheumatoid arthritis samples from a few datasets.

### 2.2. Merge and Batch Effect Removing of the Datasets

We utilized the R package inSilicoMerging [16] to merge the datasets. The before-merge and after-merge matrix are shown in Supplementary material “Merge Matrix”. Furthermore, the empirical Bayes method [17] was used to remove the batch effects. The batch-removed matrix is shown in Supplementary material “Remove_Batch”.

### 2.3. Differentially Expressed Genes (DEGs) Analysis

We utilized the R package Limma [18] to analyze the DEGs. Specifically, we first utilized the Imfit function to find the multiple linear regression of the datasets. Then, we used the eBays function to compute moderated t-statistics, moderated F-statistics, and log-odds of differential expression by the empirical Bayes moderation of the standard errors towards a common value. Finally, we acquired the differential significance of each gene. We set the fold change as 1.5 and the *p*-value as <0.05 to screen the target genes. The data are shown in Supplementary material “DEGs”.

### 2.4. Immune Cell Infiltration Analysis

To explore the infiltration of immune cells in synovium and cartilage tissues, we utilized the CIBERSORT [19] method in the R package IOBR [20] (a computational tool for immune–oncology biological research) to analyze each sample’s 22 different immune cell scores. The correlation analysis was conducted using the Pearson method. We considered *p* < 0.05 as statistically significant. The original data are shown in Supplementary material “Cell infiltration”.

### 2.5. Weighted Gene Coexpression Network Analysis (WGCNA)

We first utilized the expression matrix to calculate each gene’s median absolute deviation (MAD) and excluded 50% of the lowest MAD genes. Then, we used the goodSampleGenes method of the R package WGCNA to exclude outlier samples and genes. Subsequently, we constructed a scale-free co-expression network. Furthermore, we merged modules with a distance of less than 0.25. Module eigengene (ME) and gene significance (GS) were used to correlate clinical phenotypes and module genes. We set the MM threshold as 0.8, GS threshold as 0.1 and weight threshold as 0.1 to screen the hub genes.

### 2.6. Protein-Protein Interaction Network (PPI)

We depicted the PPI network using the online website String. (https://cn.string-db.org/ accessed on 12 November 2022) String is a free online tool that can calculate the network between specified proteins.

### 2.7. Functional and Pathway Enrichment

We utilized the Kyoto Encyclopedia of Genes and Genomes (KEGG) and gene ontology (GO) enrichment method to analyze the screened genes. For KEGG functional enrichment analysis, we adopted KEGG rest API to acquire the latest gene annotation and for GO functional enrichment analysis, we utilized the gene annotation in the R package org.Hs.eg.db (version 3.1.0). The analysis was performed using the R package clusterProfiler (version 3.14.3). We set the minimum gene set as 5, the maximum gene set as 5000 and considered *p* value < 0.05 and FDR < 0.25 statistically significant. The results are shown in the bubble diagram.

### 2.8. Characteristic Gene Screening by LASSO-Cox Regression Analysis

We utilized the R package “glmnet” to perform a regression analysis of the datasets. In this research, we set the survival time as a constant 100, “Healthy” as 0, and “OA” as 1. Thus, we could perform a regression analysis integrating gene expression and OA. The lambda score was 0.027675908746789. The original data are shown in Supplementary material “LASSO-cox”.

### 2.9. Verification of the Targets

We selected the cartilage dataset to perform a correlation analysis between risk genes and OA phenotype-related genes using the Pearson method to verify the reliability of the genes we screened. *p* value < 0.05 was considered statistically significant.

### 2.10. Statistical Analysis Software

R software (Austria) was used to calculate the statistical significance.

## 3. Results

### 3.1. Intersection of Multiple Datasets

**Figure 1:**
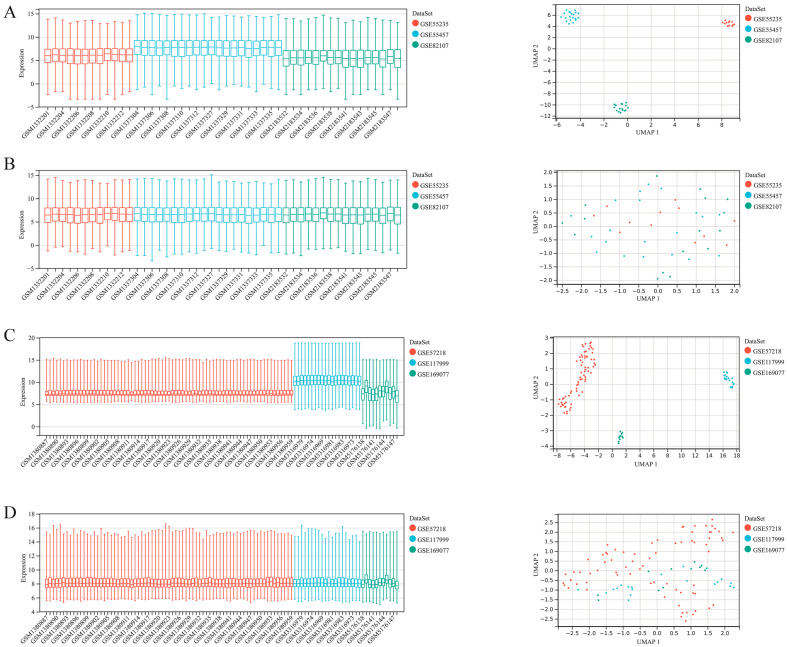
Dataset integration. The results are shown in box plots and density graphics. GSE55235, GSE55457, GSE82107: before merge (**A**), after merge (**B**), GSE57128, GSE117999, GES169077: before merge (**C**), after merge (**D**).

The datasets were obtained from GEO. GSE55235, GSE55457 and GSE82107 provided information for transcriptome sequencing of synovium tissues, while GSE57128, GSE117999 and GSE169077 provided the same for cartilage. The samples were from a healthy population and osteoarthritis patients. We utilized the R package inSilicoMerging [16] to merge the datasets. (Figure 1A,C) As is shown in the box plots and density graphic, the sample distributions of the datasets were quite different. Then, we used the empirical Bayes method [17] to adjust the batch effects of the merged datasets. (Figure 1B, D) This figure indicates that the batch effect was removed effectively. The median was on a line in the box plots, and the mean and variance were close in the density graphic. In conclusion, we successfully merged three independent datasets, thus avoiding the analysis error of using different datasets.

### 3.2. Differentially Expressed Gene Analysis

**Figure 2:**
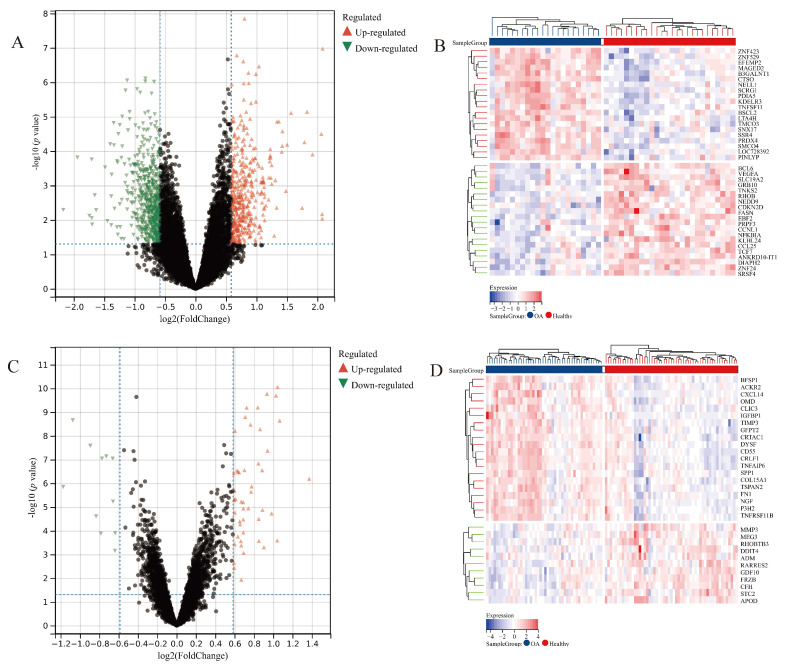
Differentially expressed gene analysis: volcano plot of the synovium dataset, (**A**); heat map of the synovium dataset, (**B**); volcano plot of the cartilage dataset, (**C**); and heat map of the cartilage dataset, (**D**).

Limma is a widely used analytical method to screen differentially expressed genes [18]. We obtained 422 up-regulated genes and 596 down-regulated genes in the synovium dataset (Figure 2A), while 50 up-regulated genes and 11 down-regulated genes were in the cartilage dataset (Figure 2B). The expressions of the top 20 genes are shown in heat maps (Figure 2C,D). These results correspond with the mainstream view that the pathological and functional changes of the articular synovium occur first, even before visible cartilage changes [21]. Thus, we selected the synovium database as the further research object in this research.

### 3.3. Immune Cell Infiltration Analysis

**Figure 3:**
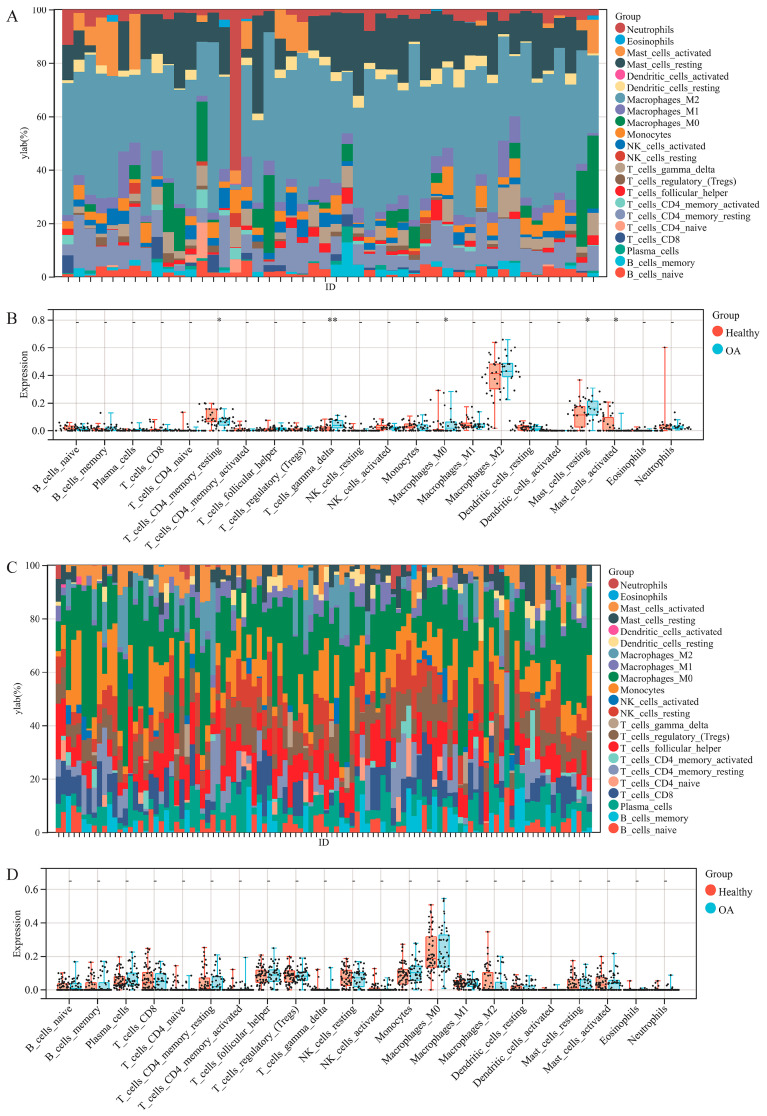
CIBERSORT immune cell infiltration analysis. Stack diagram of immune cells in the synovium dataset, (**A**). Box and scatter plot of the synovium dataset, (**B**). Stack diagram of immune cells in the cartilage dataset, (**C**). Box and scatter plot of the cartilage dataset, (**D**).

To explore the function of the immune system in osteoarthritis, we first performed immune cell infiltration analysis using the CIBERSORT method. The results of the stacked bar graphic show that the main infiltrative immune cell in both synovium and cartilage was the M2 macrophage (Figure 3A,C). Noticeably, no statistically significant changes could be observed in the cartilage tissue. However, the infiltration of γδT cells, resting mast cells and M0 macrophages increased in osteoarthritis synovium tissues while the infiltration of resting memory CD4^+^ T cells and activated mast cells was reduced (Figure 3B,D). These results suggest that varying degrees of immune cell infiltration might play a vital role in the pathogenesis of synovial inflammation in osteoarthritis.

### 3.4. WGCNA Analysis

**Figure 4:**
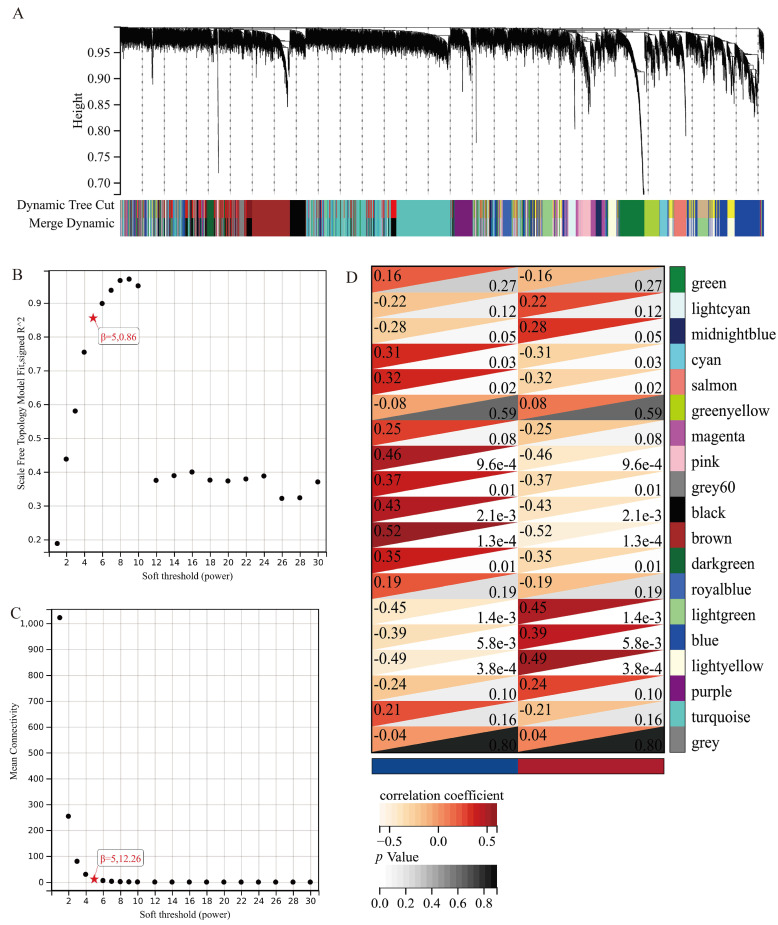
WGCNA cluster detection. Gene cluster (**A**) Independence of scale (**B**) Average connectivity (**C**) Module−phenotype correlation heat map (**D**).

We obtained 19 co-expressed modules represented by different colors (Figure 4A). Notably, the grey module was considered to not be assigned to any of the modules. The soft threshold was 5, and the scale-free topology model fit degree was 0.86 (Figure 4B). In addition, the average connectivity was 12.26 (Figure 4C). We noticed that light green, blue and light yellow were of statistically significance in all the positive correlated modules (Figure 4D). Based on these findings, we selected these three modules for further KEGG and GO functional enrichment analysis.

### 3.5. Functional and Pathway Enrichment for the Modules

**Figure 5:**
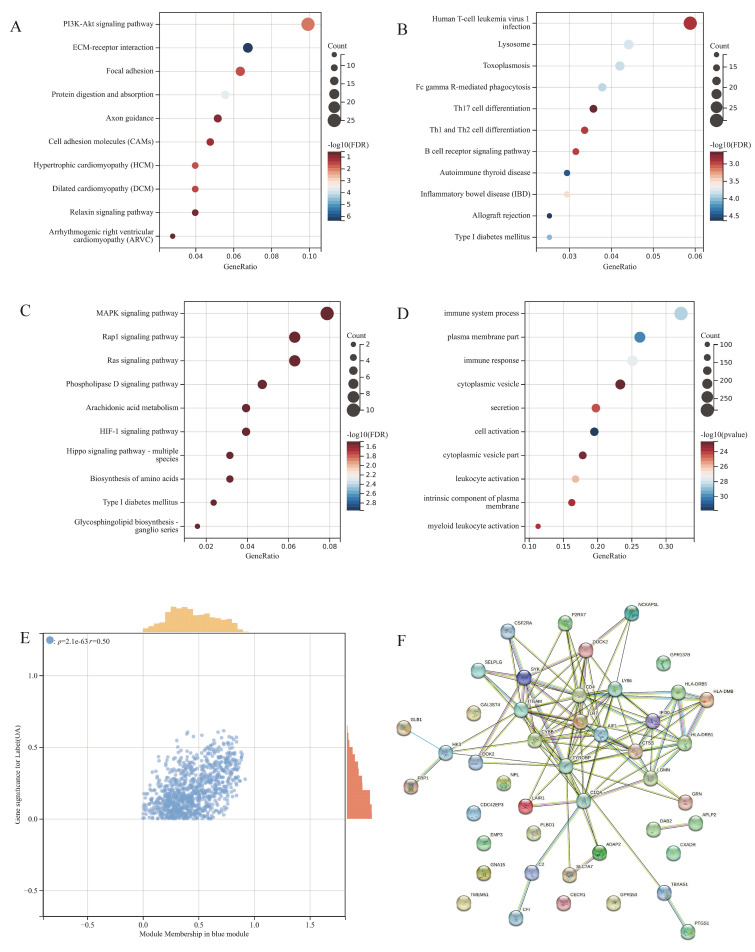
Functional and pathway enrichment and module screen: KEGG enrichment of the light-yellow module (**A**) KEGG enrichment of the blue module (**B**) KEGG enrichment of the light-green module (**C**) GO enrichment of the blue module (**D**) GS−MM correlation scatter plot of the blue module (**E**) PPI network of hub genes (**F**).

We performed KEGG enrichment for each module. The light-yellow module is involved mainly in the PI3K-Akt signaling pathway and ECM-receptor interaction (Figure 5A). The enrichment of the blue module consists mainly of human T-cell leukemia virus 1 infection, Th1, Th2, Th17 cell differentiation, B cell receptor signaling pathway, autoimmune thyroid disease and inflammatory bowel disease (Figure 5B), which indicate that the blue module is highly related to the innate immune system. The result of the enrichment of the light green module is of no statistical significance (Figure 5C). Therefore, we selected the blue module as the object of further research. We then performed GO enrichment specific to the blue module (Figure 5D). The functions with the most statistical significance were closely related to the immune system process and immune response. The scatter plot of the blue module exhibited a satisfactory linear correlation between the clinical phenotype and the blue module genes (Figure 5E). We then extracted the hub genes of the blue module and drew a PPI network, which indicated that most of the hub genes interacted with each other (Figure 5F). These results suggest that the blue module is of great potential as the objective module for further characteristic gene identification.

### 3.6. Machine Learning for Prediction of High-Risk Genes

**Figure 6:**
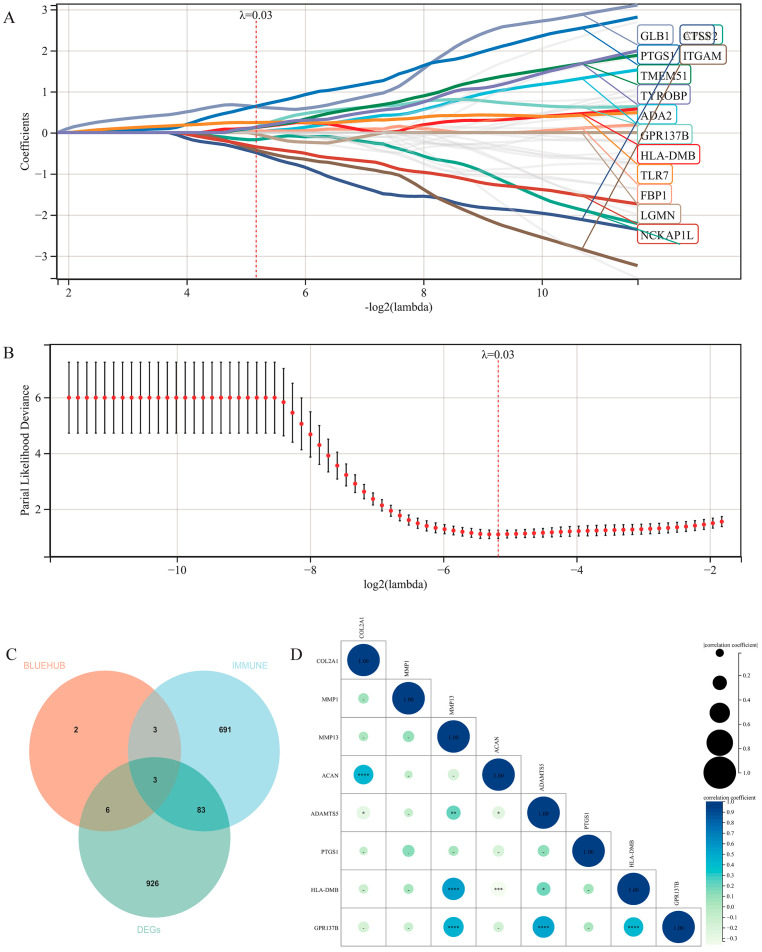
LASSO-cox regression analysis. Coefficient distribution diagram (**A**) Lambda selection (**B**) The intersection of DEGs, immune-related genes and characteristic genes (**C**) Correlation analysis of target genes and OA phenotype-related genes (**D**). * *p* < 0.05, ** *p* < 0.01, *** *p* < 0.001, **** *p* < 0.0001.

We utilized the LASSO algorithm to identify the characteristic genes of the 39 hub genes (Figure 6A,B). We obtained 14 characteristic genes (shown in Supplementary material “LASSO-cox result”) and intersected them with the DEGs and recognized immune-related genes [18]. The final intersection consists of three genes, which are PTGS1, HLA-DMB and GPR137B (Figure 6C). Then, we performed a correlation analysis of the risk genes and recognized genes related to osteoarthritis phenotypes (Figure 6D). Noticeably, the co-expression of MMP13 and these three genes are highly relevant and statistically significant. However, the expression of MMP1 is of no obvious relation with these three genes.

## 4. Discussion

Osteoarthritis (OA) is a disabling disease affecting millions of people worldwide [1]. The factors of OA are manifold and complicated and have not been clarified yet [22]. Extensive low-grade inflammation has been recognized as a critical mediator in the progression of OA [23]. This differs from high-grade inflammation in rheumatic arthritis [24]. Biological therapies that successfully block the inflammation cytokines in RA, such as anti-IL1β, exhibited no promising prospect in the clinical management of OA [25]. Thus, it is urgent to clarify the potential mechanisms of OA to provide new approaches in drug development.

With the rapid development of computer science, advanced bioinformatic analytic methods have been applied in the identification of disease markers and decipherment of pathological mechanisms in osteoarthritis. Since the debut of RNA-sequencing (RNA-seq) technology more than a decade ago [26], it has been an indispensable tool in all kinds of aspects in the genomics field [27]. Differential gene expression (DEG) analysis is the most prominent application of the RNA-seq database [28]. Weighted correlation network analysis (WGCNA) is an R package devised for identifying gene clusters that consists of highly-correlated genes [29]. Based on these clusters, further application of KEGG and GO enrichment analysis allows researchers to focus on the biological function each cluster represents. For interested clusters, a machine learning method-LASSO regression analysis is the optimal option to reduce the dimensionality and screen out the most characteristic genes [30]. The innate system has been recognized to play a vital role in osteoarthritis [8,31]. Consistent activation of pattern-recognition receptors (PRRs) and damage-associated molecular patterns (DAMPs) produces prolonged inflammation [32]. Aging enhances the alterations of the innate system, which is termed “inflamm-aging” [33]. Macrophages and T cells are reported to be the primary immune cell group in the synovium and cartilage [8]. In vivo studies demonstrated that accumulation and activation of macrophages existed widely in OA patients’ synovia. The activation of macrophages strongly correlates with osteophytes, joint narrowing and knee pain [34]. However, the specific work pattern of the immune cells remains to be clarified.

Identifying potential disease markers of osteoarthritis has been a research hotspot in different aspects. However, most reports only utilized limited, even single, datasets [35,36,37]. We believe that merging datasets from different sources could result in a more persuasive conclusion. In this research, we integrated multiple datasets from GEO, which could effectively eliminate the batch difference of studies worldwide. The differentially expressed gene analysis found that the amount of DEGs in synovium outpaces that of cartilage. Under a 1.5-fold change, the amount of DEGs (422 up-regulated genes and 596 down-regulated genes) in the synovium is nearly 20 times as high as the amount in the cartilage (50 up-regulated genes and 11 down-regulated genes).

Then, we performed immune cell infiltration using CIBERSORT bioinformatics analysis to determine whether the immune cells infiltrated differently in tissues from healthy people or OA patients. Unexpectedly, immune cell infiltration in the OA and healthy cartilage exhibited no noticeable difference. Han et al. reported significantly different immune cell infiltration in osteoarthritis, specifically the infiltration of T cells and cytotoxic lymphocytes [4]. We reviewed the datasets selected in the report and found that they not only incorporated the datasets including articular cartilage but also the meniscus. However, we insist that meniscus lesions are a consequence as osteoarthritis develops into its terminal stage [38]. Hence, we suppose that in the research concerning identifying risk factors, it is inappropriate to include the meniscus.

Research targeting immune cell infiltration in the synovium has seldom been reported. What attracted us most was the significant increase in γδT cells in the inflammatory synovium tissues. The γδT cell plays a vital role in the innate immune system [39]; they mainly develop in the thymus and exert major histocompatibility complex (MHC) unrestricted antigen recognition [40]. Activated γδT cells participate in the innate immune process via producing inflammatory mediators [41], which might explain the crucial condition of the inflammation response existing widely in joints with varying degrees of osteoarthritis. Based on these results, we made a reasonable assumption that the immune system might exert its function primarily through the synovial tissues.

Another noteworthy point is that even though the expression of the immune cells in chondrocytes is of no significant difference, the activation of the specific immune cell is difficult to analyze using transcriptome sequencing datasets. We noticed that the macrophage is the prominent cell type in cartilage, and whether it is active in disease progression needs to be researched further. Considering the proinflammatory and anti-inflammatory effects of the different statuses and kinds of macrophages [42], experimental methods are critical concerning these cells.

To explore the correlation between co-expression genes and the clinical phenotype in healthy and OA synovium tissues, we performed WGCNA analysis. In this research, we discuss the gene modules mainly positively correlated with synovial inflammation. The KEGG enrichment of the three most significant modules indicated that the blue module is highly relevant to the immune system. Subsequent GO enrichment also verified this result. We screened the hub genes in the blue module as the candidate risk genes.

The LASSO-cox method was utilized to perform regression analysis to acquire the optimization model [43]. We acquired 11 characteristic genes, ADA2, APLP2, CTSS, FBP1, GLB1, GPR137B, HLA-DMB, ITGAM, LGMN, NCKAP1L, PTGS1, TLR7, TMEM51 and TYROBP. Then, we intersected the DEGs and characteristic genes, recognizing immune-related genes [44]. We finally acquired three risk genes, PTGS1, HLA-DMB and GPR137B. Highly-expressed PTGS1 levels in the synovium promote migration and invasion and inhibit the cell apoptosis of inflammatory synovial cells [45]. HLA-DMB is a prognostic factor in rheumatoid arthritis [46]; however, its role in the processing of osteoarthritis has not been clarified yet. In a genome-wide association study, GPR137B was reported to be correlated with hereditary susceptibility for rheumatoid arthritis [47]. Thus, these three genes could be potential targets in future research on osteoarthritis.

To verify the function of the target genes in osteoarthritis, we performed a matrix correlation analysis of the risk genes and OA phenotype-related genes in the cartilage datasets. Interestingly, the result indicates that the risk genes are highly relevant to MMP13 expression and have remarkable statistical significance. This result is consistent with a theory that chondrocytes probably secrete MMP13 in the cartilage. In addition, the three genes did not correlate with MMP1, which is secreted mainly by synovial cells [48].

This research has a few limitations that have the potential to be research goals in the future. Biological verification must be performed to confirm our results, at least in an animal model. Additionally, we noticed that γδT cell infiltration in the synovial tissue was not researched thoroughly. An establishment of a reliable synovium–cartilage axis model is urgently needed.

Collectively, our research analyzed the immune-related genes in the synovium and cartilage tissues of the healthy population and OA patients. First, we demonstrated that the transformation of immune cell infiltration might mainly exist in the synovium and not the cartilage. These results support the opinion that changes in the synovium might appear before cartilage erosion. In addition, we provided three genes as potential disease markers and future drug development targets.

## Data Availability

All data presented in this study are available from the corresponding author upon reasonable request.

## Supplementary Materials

The following supporting information can be downloaded at: https://www.mdpi.com/article/10.3390/jpm13020367/s1. Supplementary File S1: Merge_Matrix, Remove_Batch, DEGs, cell infiltration and LASSO_cox.
